# The Fee for Testifying as Expert

**Published:** 1869-01

**Authors:** 


The Fee for Testifying as Expert.—Dr. Bebee, of Chicago, was called to testify in a case before the United States Circuit Court at Chicago, not long since, as a medical expert. He refused to testify unless he received fees as an expert, to the amount of $25. The Judge decided he was right and the fees were paid.

